# CD3^+^CD4^-^CD8^-^ (Double-Negative) T Cells in Inflammation, Immune Disorders and Cancer

**DOI:** 10.3389/fimmu.2022.816005

**Published:** 2022-02-10

**Authors:** Zhiheng Wu, Yu Zheng, Jin Sheng, Yicheng Han, Yanyan Yang, Hongming Pan, Junlin Yao

**Affiliations:** Department of Medical Oncology, Sir Run Run Shaw Hospital, College of Medicine, Zhejiang University, Hangzhou, China

**Keywords:** double-negative T cell, inflammation, immune disorders, cancer, immunoregulation, helper function, tumor microenvironment, immunotherapy

## Abstract

The crucial role of CD4^+^ and CD8^+^ T cells in shaping and controlling immune responses during immune disease and cancer development has been well established and used to achieve marked clinical benefits. CD3^+^CD4^-^CD8^-^ double-negative (DN) T cells, although constituting a rare subset of peripheral T cells, are gaining interest for their roles in inflammation, immune disease and cancer. Herein, we comprehensively review the origin, distribution and functions of this unique T cell subgroup. First, we focused on characterizing multifunctional DN T cells in various immune responses. DN regulatory T cells have the capacity to prevent graft-versus-host disease and have therapeutic value for autoimmune disease. T helper-like DN T cells protect against or promote inflammation and virus infection depending on the specific settings and promote certain autoimmune disease. Notably, we clarified the role of DN tumor-infiltrating lymphocytes and outlined the potential for malignant proliferation of DN T cells. Finally, we reviewed the recent advances in the applications of DN T cell-based therapy for cancer. In conclusion, a better understanding of the heterogeneity and functions of DN T cells may help to develop DN T cells as a potential therapeutic tool for inflammation, immune disorders and cancer.

## Background

The human immune system plays an indispensable role in the maintenance of physical homeostasis. There are two major components of the immune system: the innate and adaptive immune systems. The overwhelming majority of adaptive immune cells are TCR alpha and beta (αβ) positive with the expression of either CD4 or CD8, while double-negative (DN) T cells, which are CD3 positive but lack both CD4 and CD8 coreceptors, can express either TCRaβ or TCR gamma and delta (γδ) and do not express natural killer (NK) T cell markers. Although representing a small subpopulation of approximately 3-5% of T lymphocytes in peripheral blood (1, 2), DN T cells have been found to possess both innate and adaptive immune functions, differing from conventional CD4^+^ and CD8^+^ T cells. It is worth noting that we mainly focus on the rare TCRαβ^+^ DN T cell subset throughout the review.

Though DN T cells have not been classified by certain phenotypes, a recent study revealed subgroups of naïve and active TCRαβ^+^ DN T cells in mice by using single-cell RNA sequencing. In that study, naïve DN T cells were defined as resting/helper/intermediate/cytotoxic/innate T cells with representative transcriptomic signatures, and active DN T cells were divided into cytotoxic and proinflammatory subgroups (3). DN T cells have also been proven to share functions with various types of differentiated effector T cell subsets in different settings (3). As a subset of regulatory T (Treg) cells, DN T cells can prevent graft-versus-host disease (GVHD) and have therapeutic value for autoimmune disease, depending on their regulatory effects on CD8^+^ T cells, CD4^+^ T cells and B cells (4–6). T helper (Th)-like DN T cells secrete cytokines, including interleukin (IL)-4, IL-17, interferon-γ (IFN-γ) and tumor necrosis factor-α (TNF-α), and play a similar role to CD4^+^ Th cells in infection and autoimmune disease (7–9). Cytotoxic DN T cells produce IFN-γ, perforin and granzyme B, mediating the killing effect in hematologic malignancies and solid tumors (10, 11). Here, we comprehensively review the different functions and diverse roles of DN T cells in inflammation, immune disorders, cancer development and immunotherapy. We further uncover the potential molecular mechanisms behind these disease models and try to compare multifunctional DN T cells with traditional CD4^+^ and CD8^+^ T cells in certain disease states. This review aims to uncover the complex roles of DN T cells and broaden the knowledge of the immune system.

## Characteristics and Distribution of DN T Cells

Approximately 95% of T cells express TCRαβ and 5% of T cells express TCRγδ in humans. Almost all CD8^+^ T cells and CD4^+^ T cells are TCRαβ^+^ T cells, while DN T cells contain both TCRαβ^+^ T cells and TCRγδ^+^ T cells in different proportions according to various studies, and the proportions seem to be affected by extraction, purification and expansion methods. Generally, TCRαβ^+^ cells participate in the adaptive immune response, whereas TCRγδ^+^ T cells might participate in both innate and adaptive immunity (12). Therefore, DN T cells are considered members of both innate and adaptive immune systems. Here, we mainly discuss the characteristics and distribution of rare TCRaβ^+^ DN T cells.

TCRαβ^+^ DN T cells comprise 1-3% of mature peripheral CD3^+^ T cells in both human and mouse models (1, 4). In addition to peripheral blood, DN T cells are observed in secondary lymphoid organs and various tissues, including the intestine, liver, lung, skin and genital tract, and these cells are identified as tissue-resident DN T cells. Tissue-resident DN T cells have distinct phenotypes under different physiological and pathological settings. TCRαβ^+^ DN T cells comprise one-third of normal intraepithelial lymphocytes in the murine gut, and gut-resident DN T cells have phenotypes similar to those of peripheral DN T cells in mice (13, 14). A significant proportion of hepatic DN T cells is found in normal murine and human liver tissue, and DN T cells accelerate murine acetaminophen toxicity by expressing the activation marker CD69 and Fas ligand (FasL) (15, 16). DN T cells were reported to reside as preactivated tissue resident memory-like cells in the lung parenchyma, expressing the memory markers CD44, CD11a and CD103, and the cytotoxic effector molecule FasL; therefore, resident memory DN T cells expand rapidly to protect against influenza A virus infection (17). Although mammalian kidneys do not conventionally harbor resident lymphocytes, DN T cells are an important subset of the resident TCRαβ^+^ T cells in normal murine and human kidneys (18, 19). In addition, DN T cells can migrate from peripheral blood to organs, as circulating DN T cells have been shown to infiltrate the salivary gland, skin and kidneys and to be involved in the immunopathogenesis of Sjögren’s syndrome (20). Thus, TCRaβ^+^ DN T cells are widely distributed as both tissue-resident T cells and circulating cells, participating in a series of physiological and pathological processes.

## The Origin of DN T Cells

Traditionally, T lymphocytes originate from progenitors in bone marrow and then migrate to the thymus for maturation, selection and subsequent transport to the periphery. Previous studies on both transgenic and wild-type mouse models provide us with scientific clues about the origin and developmental pathway of DN T cells. Studies show that DN T cells come from a legitimate group of thymocytes in particular stages in the thymus, similar to CD4^+^ and CD8^+^ T cells; alternatively, DN T cells can also arise from peripheral mature T cells due to an intrinsic defect (21).

### Thymic Origin of DN T Cells

During T cell development in the thymus, several specific stages have been suggested to give rise to TCRαβ^+^ and TCRγδ^+^ DN T cells in mouse models.

T lymphocytes in later DN stages transform into either TCRαβ^+^ or TCRγδ^+^ DN thymocytes, depending on TCR signal strength. During the DN3 stage, high TCR strength makes TCRγδ^+^ DN thymocytes remain “double-negative”, whereas DN cells with pre-TCRs follow the αβ lineage fate followed by single-positive (SP) and double-positive (DP) stages in sequence (22–26). However, Chapman et al. described that sex steroid-activated thymic mast cells could induce both TCRαβ^+^ and TCRγδ^+^ DN thymocytes to leave the thymus instead of transit through the next stages, suggesting that peripheral TCRαβ^+^ DN T cells can also directly come from the DN stage in the thymus (27).

**Figure 1:**
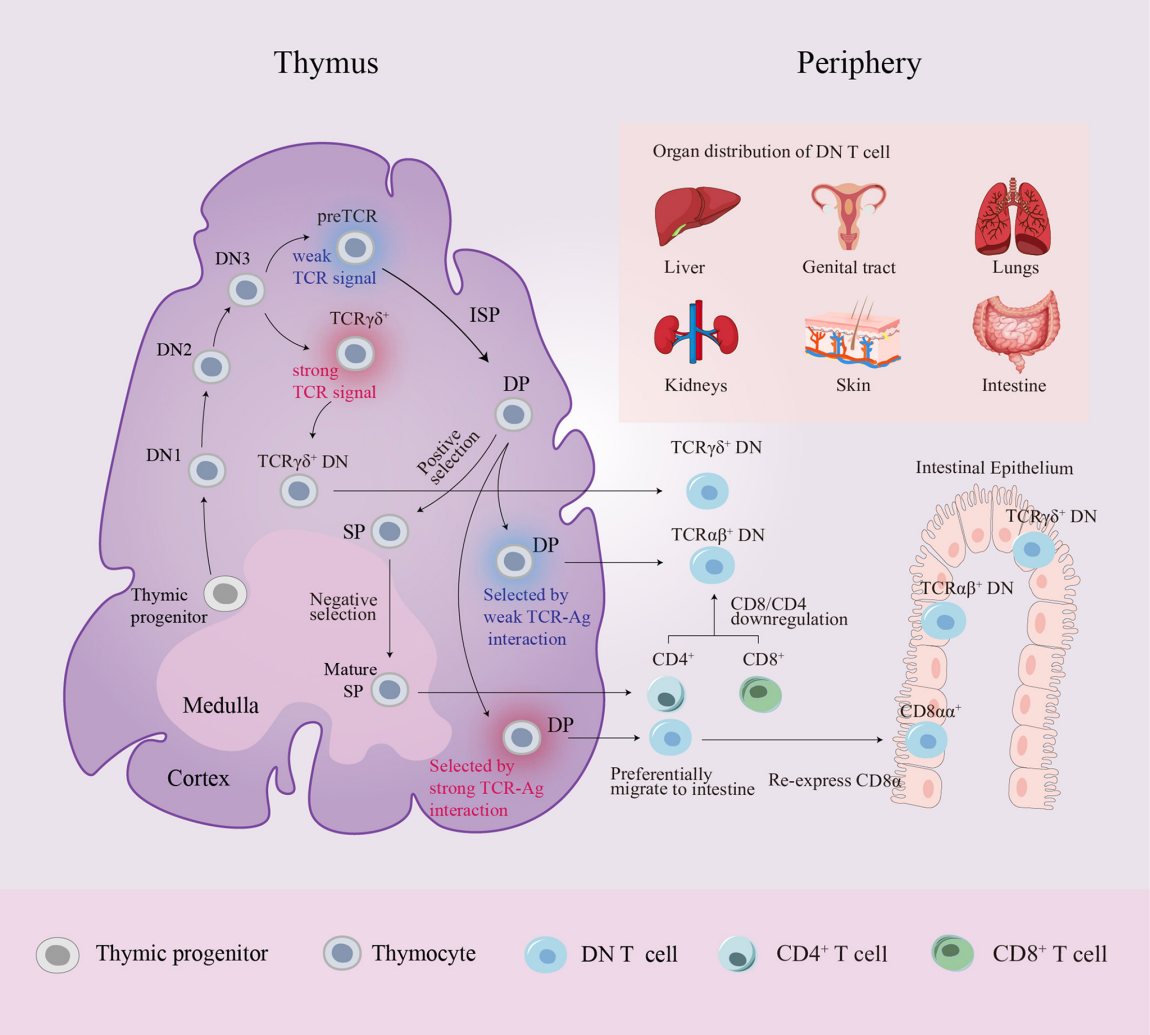
Schematic diagram of DN T cell origin and distribution. Progenitor cells from the bone marrow colonize the corticomedullary junction of the thymus and generate the T cell lineage. In the DN3 stage, generally, high TCR strength makes TCRγδ^+^ DN thymocytes remain “double-negative”, whereas low TCR strength induces DN thymocytes with pre-TCRs to follow the αβ lineage fate. A small number of DP thymocytes differentiate into two distinct mature DN thymocyte subsets rather than entering the late SP stage: DN thymocytes selected by very strong TCR-MHC interactions preferentially migrate to the intestine where they re-express CD8α and are sequestered as CD8αα^+^ intraepithelial lymphocytes, while DN thymocytes selected by very weak TCR-MHC interactions become precursors to peripheral TCRαβ^+^ DN T cells. In peripheral circulation, CD4^+^/CD8^+^ T cells can also convert into DN T cells by downregulating CD4/CD8 expression. Peripheral DN T cells are localized in various organs in mice and humans. αβ, alpha and beta; Ag, antigen; DN, double-negative; DP, double-positive; γδ, gamma and delta; ISP, immature single-positive; MHC, major histocompatibility complex; SP, single-positive; TCR, T cell receptor.

To determine the cellular origin of TCRαβ^+^ DN T cells, CRE/YFP transgenic mouse models were used to conduct fate mapping experiments, proving that DN T cells can be traced back to the DP stage (28, 29). Interactions between DP thymocytes and high-affinity antigen (Ag) presented by cortical epithelial cells result in immunoregulatory TCRαβ^+^ DN T cell differentiation through the downregulation of the expression of CD4 and CD8 (30). In addition to producing SP T cells, the thymus can also generate DN thymocytes during the DP stage. Previous studies have demonstrated that the diversion from immature DP thymocytes to mature DN thymocytes requires strong TCR-mediated, major histocompatibility complex (MHC) -specific thymic selection signals. These DP thymocytes that received strong signals undergo developmental diversion and differentiate into TCRαβ^+^ DN thymocytes that preferentially migrate to the intestine where they re-express CD8α and are sequestered as CD8αα intraepithelial lymphocytes (31, 32). Recently, Rodríguez-Rodríguez et al. and Collin et al. provided evidence that the mature TCRαβ^+^ DN thymocyte pool also includes precursors to peripheral TCRαβ^+^ DN T cells distinct from other unconventional T cells. In contrast to TCRαβ^+^ CD8αα^+^ intraepithelial lymphocytes, peripheral TCRαβ^+^ DN T cells have been selected by very weak TCR-self-Ag interactions through the DP stage, and the selection proceeds in an MHC-independent setting. Consequently, these TCRαβ^+^ DN T cells lack MHC restriction (28, 29) (**Figure 1**).

### Extrathymic Origin of DN T Cells

Several lines of convincing evidence have uncovered unusual thymus-independent sources of TCRαβ^+^ DN T cells, which can be further roughly divided into either CD8^+^ T cell-derived DN T cells or CD4^+^ T cell-derived DN T cells.

Due to the accumulation of TCRαβ^+^ DN T cells in mice with defective Fas or FasL, Fas or FasL knockout mice are ideal models to study the origin of TCRαβ^+^ DN T cells (33). When comparing Fas-deficient and Fas-intact mouse models after peripheral TCRαβ^+^ cell deletion induced by specific peptide administration, DN T cells are significantly increased only in Fas-deficient models with the corresponding downregulation of CD8^+^ T cells, indicating the possibility of transformation of DN T cells from TCRαβ^+^CD8^+^ cells (34). Autoimmune lymphoproliferative syndrome (ALPS) is characterized by the accumulation of DN T cells in the periphery. In an analysis of CDR3 sequences of matching clonotypes, DN T cells and CD8^+^ T cells showed a significant sharing of CDR3 sequences from selected Vbeta-Jbeta transcripts from ALPS patients. Analysis of CDR3 length distribution revealed that both DN T cells and CD8^+^ T cells have a skewed TCR repertoire, with oligoclonal expansions throughout most of the V beta families in ALPS patients (35, 36). A recent study based on lpr mouse model with splenic marginal zone macrophage deficiency further demonstrated that exposure to apoptotic cell debris induces the loss of CD8 but not CD4 expression, which in turn provokes the generation of DN T cells (37). In addition, normal human TCRαβ^+^ DN T cells were also reported to differentiate from peripheral CD8^+^ T cells and then gain proinflammatory capacity (38). Therefore, these data strongly support the CD8^+^ T cell origin of TCRαβ^+^ DN T cells.

Peripheral CD4^+^ progenitors of TCRαβ^+^ DN T cells have also been demonstrated in various studies. Ford et al. found DN T cells in both thymectomized mice and CD8-deficient mice, indicating that DN T cells can develop independently of the thymus or CD8^+^ T cell precursors (39). Soon after that study, CD4 gene silencing was reported to convert mature murine peripheral CD4^+^ T cells into MHC II-restricted DN Treg cells, and the converted DN T cells have unique cell surface markers and gene profiles that are different from CD4^+^ T cells (40). A recent study also demonstrated that immature bone marrow dendritic cells can induce high levels of DN Treg cells from CD4^+^CD127^hi^CD25^-^ T cells (41). More recent studies have shown that tumor necrosis factor receptor superfamily member 4 (Ox40), a costimulatory molecule highly expressed on DN Treg cells, plays a crucial role as a potent regulator of the proliferation and survival of CD4^+^ T cell-derived DN Treg cells (42–44). Notably, human Fas-deficient DN T cells share CDR3 sequences with both CD4^+^ and CD8^+^ terminally differentiated effector memory cells, providing novel evidence that human DN T cells can be derived from not only CD8^+^ T cells but also CD4^+^ T cells (45).

In addition to the thymus progenitors, peripheral CD8^+^ and CD4^+^ progenitors of TCRαβ^+^ DN T cells are also vital developmental precursors of DN T cells. Interestingly, DN T cells that arise outside the thymus have different characteristics from DN T cells that developed in the thymus and their precursor CD4^+^ or CD8^+^ T cells. Further investigation into the molecular mechanisms of the developmental pathways may provide more information about this unique subset of T cells.

## Immunoregulatory DN T Cells Guard the Body Against Aggressive Immune Responses

Treg cells are critical for maintaining immune tolerance and preventing autoimmunity. DN Treg cells have attracted attention as novel Treg cells. As the origin of DN T cells is based on various mouse models without a specific transcript profile, unlike naturally occurring CD4^+^CD25^+^Foxp3^+^ Treg cells, DN Tregs are not a clear distinct lineage and can only be defined by their suppressive function.

### DN Treg Cells Regulate the Immune Response in Antigen-Specific and Non-Antigen-Specific Manners

DN Treg cells have been shown to regulate alloreactive, xenoreactive and autoreactive T cells in various mouse models and play therapeutic roles in GVHD and autoimmune disease. Ag-stimulated murine DN Treg cells have been shown to suppress activated CD8^+^ and CD4^+^ T cells, B cells and dendritic cells in various mouse models, partly relying on Ag-specific and non-Ag-specific cell contact (4–6, 46). Allo-Ag-specific cell contact requires DN Treg cells to obtain allo-Ag from donor-derived antigen presenting cells through trogocytosis, resulting in the recognition of Ag- specific syngeneic CD8^+^ T cells and subsequently the suppression of allogeneic immune responses (47, 48). As DN T cells are sufficient to induce skin and cardiac allograft survival (4, 49), they should be able to inhibit GVHD caused by syngeneic CD8^+^ and CD4^+^ T cells. In addition to allo- or xeno-MHC/TCR interactions, peptide-self MHC complexes also contribute to the TCR-specific activation of DN Treg cells (50). Similar to CD8^+^ T cells, memory-like self Ag-experienced DN T cells have the potential to expand and differentiate into Treg cells, which eliminate autoreactive CD8^+^ T cells through the Fas-FasL interaction (51). Indeed, DN Treg cells can prevent the onset of and provide long-lasting protection against type 1 diabetes and GVHD (52–56). Moreover, a recent clinical trial suggested that Ag-experienced DN Treg cells could inhibit the development of chronic GVHD by eliminating self-reactive B cells in a cohort of allogeneic hematopoietic stem cell transplantation recipients (57). In conclusion, these findings indicate the existence of a novel subset of Treg cells that can prevent GVHD and have therapeutic value for autoimmune disease.

### Molecular Characterization of DN Treg Cell Immunoregulation

The cytotoxic effect of DN Treg cells on CD8^+^ T cells and CD4^+^ T cells is mainly mediated by the Fas-FasL pathway, the perforin-granzyme pathway and the cytokine IFN-γ. In addition, other molecules expressed on DN Treg cells also participate in the regulatory network. The FcRγ subunit is present in the TCR complex of DN Treg cells and plays a role in mediating TCR signaling in the suppression of CD8^+^ T cells (58); chemokine receptor CXCR5 promotes DN Treg cell migration (49, 59); Ly-6A enhances DN Treg cell-mediated cytotoxicity with a putative role in cell adhesion (60, 61); and the coinhibitory receptor Lag-3 contributes to Ag recognition by DN Treg cells through the engagement of MHC II (62, 63). Natural-killer group 2, member D (NKG2D) positive DN T cells express higher levels of granzyme B than their NKG2D^-^ counterparts, promoting B cell apoptosis and inhibiting B cell proliferation (41). In addition, the cytokine IFN-γ further enhances FasL-mediated cytotoxicity of DN T cells, while IL-10 inhibits DN T cell expansion and activation (64–66). More recently, Ox40 was found to participate in DN Treg cell proliferation, survival and regulatory function (43) (**Table 1**). Therefore, a complex molecular network exhibits a critical role in regulating DN Treg cell function.

**Table 1:** Regulatory DN T cell in immune response.

Mouse model or human	DN T cell phenotype	Mechanism of action	Target cell	Reference
2C_F1_ (H-2^b/d^, L^d-^, anti-L^d^ 1B2-TCR^+^) transgenic mice	TCRαβ^+^CD25^+^CD28^-^CD30^+^CD44^-^	Acquire peptide-MHC from APC *via* trogocytosis of MHC class I, Fas/FasL pathway	Alloreactive CD8^+^ T cell	(4–6)
	Ly-6A^+^	Ly-6A dependent, Ly-6A expression inhibited by IL-10	Syngeneic CD8^+^ T cell	(60)
	CTLA-4^+^	Downregulation of CD80/CD86 expression on DC, Fas/FasL pathway	DC	(67)
H-2^b/d^ 2C TCR transgenic mice	TCRαβ^+^CD25^-^CD69^-^CD44^+^ IL-2Rβ^+^Ly-6C^+^1B11^+^	TCR-Ag interaction, Fas/FasL pathway	Autoreactive CD8^+^ T cell	(51)
B6 mice	TCRαβ^+^B220^+^CD25^+^CD28^-^ CD40L^-^CTLA-4^-^	Ag-dependent activation, perforin/granzyme mediated bystander effect	Syngeneic B cell	(6)
Diabetes-resistant B10.Br 3A9/Diabetes-prone NOD.*H^2k^* 3A9 mice	CD107a^+^	Perforin/granzyme, Ag-specific	B cell	(65)
Diabetes-prone NOD mice	TCRαβ^+^CD3^+^CD28^+^CD69^+^ CD25^low^Foxp3^-^iCTA-4^-^	Differentiate preferentially to IL-10 producing Treg cell in Th2 environment	-	(52)
Allogeneic heart grafts from CB6F1 mice transplanted into 2C_F1_ transgenic mice	CXCR5^+^	CXCR5/CXCL13 interaction promotes DN T cell migration	-	(59)
Healthy human	TCRαβ^+^CD25^low^CD28^low^ CD95^high^CTLA-4^-^	Acquire peptide-MHC from APC, cell-cell contact	Activated CTL	(1)
	-	TCR-dependent inhibition, cell-cell contact	CD4^+^ T Cell	(68)

αβ, alpha and beta; Ag, antigen; APC, antigen presenting cell; CTL, cytotoxic lymphocyte; CTLA-4, cytotoxic T-lymphocyte-associated protein 4; CXCL, chemokine; CXCR, chemokine receptor; DC, dendritic cell; DN, double-negative; γδ, gamma and delta; IL, interleukin; MHC, major histocompatibility complex; NOD, non-obese diabetic; TCR, T cell receptor; Th2, T-helper type 2; Treg, regulatory T.

The functions of DN Treg cells have been deeply investigated in mouse models, and whether human DN T cells have distinct functions has also been studied. Studies have shown that human DN T cells have mechanisms and phenotypes similar to those of their murine counterparts, as the suppression effect is also TCR dependent. Despite sharing Ag-specific recognition with their murine counterparts, human DN T cells probably keep CD8^+^ effector T cells in cell cycle arrest rather than eliminating them (1, 68, 69). Moreover, human DN T cells inhibit the immune response caused by CD4^+^ effector T cells by abrogating Akt/mTOR signaling in a non-Ag-specific manner (70, 71). Therefore, the human DN T cell subset also exhibits remarkably potent immunosuppressive potential, paving the way for further clinical application.

## T Helper-Like DN T Cells Regulate the Immune Response as a Double-Edged Sword

Despite having a regulatory function similar to that of naturally occurring Treg cells, DN T cells also secrete cytokines (IL-4, IL-17, IFN-γ and TNF-α) to exert Th function, so these subsets are identified as Th-like DN T cells. Th-like DN T cells exhibit either protective or pathologic functions in different infections.

### DN T Cells Participate in Inflammation and Virus Infection

In certain infectious disease models, DN T cells fit into a highly activated T cell subpopulation, producing cytokines for activation of immune responses and protection against infections. Mechanistically, DN T cells are restricted by either MHC I or MHC II molecules, which influences the effector cytokines they produce, including IL-17, TNF, IFN-γ and granzyme B, and DN T cells from healed mice have an effector memory phenotype similar to CD95^+^CD62L^-^ T cells. Furthermore, DN T cells participate in maintaining both innate and adaptive responses, modulating the functions of macrophages, CD8^+^ T cells and B cells (72–75). In the periphery of Leishmania-infected patients, TCRαβ^+^ DN T cells account for approximately 75% of all DN T cells, with the remainder expressing TCRγδ. While TCRαβ^+^ DN T cells present a proinflammatory cytokine profile, TCRγδ^+^ DN T cells are likely to work as a negative regulator of IL-10 production (76). Furthermore, DN T cells from Leishmania-infected mice predominantly express TCRαβ. The study directly proved that these DN T cells contribute to primary and secondary immunity restricted by MHC II. Leishmania-reactive TCRαβ^+^ DN T cells might activate macrophages and subsequently increase macrophage antiparasitic capacity (73). In addition, TCRαβ^+^ DN T cells are a major responding T cell subset in the lungs of mice, protecting against Francisella tularensis infection by producing IL-17A and IFN-γ (72). Two groups of DN T cells in Mycobacterium tuberculosis patients show different patterns. Overall, the proportions of TCRαβ^+^ DN T cells positively correlate with the severity of the disease, while the numbers of TCRγδ^+^ DN T cells are not different between infected and healthy populations. The inflammatory components of TCRαβ^+^ and TCRγδ^+^ DN T cells are maintained among individuals with nonsevere disease, while enhanced IL-10 production by TCRγδ^+^ DN T cells is present in more advanced disease, which may cause disease progression (77). Researchers have also studied the roles of DN T cells in virus infection. DN T cells from natural nonhuman hosts of simian immunodeficiency virus exhibit an effector memory phenotype, and the presence of Th-like DN T cells is associated with suppression of clinical progression of disease induced by simian immunodeficiency virus because these cells produce Th1, Th2 and Th17 cytokines (7, 8). However, DN T cells (not clearly divided into TCRαβ^+^ or TCRγδ^+^ subsets) play functionally distinct roles in human immunodeficiency virus (HIV) infection since they used to be described as Treg cells as well as a reservoir for HIV-infected cells that can lead to the reappearance of HIV infection (66, 78). These controversial results demonstrate that the distinct functions of DN T cells depend on the specific setting.

### Proinflammatory DN T Cells Attack the Body in Certain Autoimmune Disease

In addition to Th17 cells, TCRαβ^+^ DN T cells have also been implicated in the pathogenesis of autoimmune disease. The accumulation of DN T cells has been proven to positively correlate with the disease activity of ALPS and systemic lupus erythematosus (SLE). DN T cells in ALPS exhibit a remarkable uniform phenotype that is neither effector nor memory, characterized by B220, CD27 and CD28 expression and high IL-10 production (35, 79). In lupus-prone mice, similar to the case for Th17 cells, the IL-23/IL-17 axis plays an important role in the pathogenicity of DN T cells, as these cells lead to systemic inflammation and organ damage in SLE by producing the inflammatory cytokine IL-17 and inducing anti-DNA autoantibody production (9, 37, 80). SLE-associated nephritis is a common and serious complication in SLE patients, and IL-17-producing DN T cell infiltration is positively correlated with kidney disease progression in SLE patients and murine lupus models, suggesting that DN T cells might be a biomarker as well as a therapeutic target for lupus nephritis (9, 81–83). Similarly, DN T cells may also be centrally involved in the pathogenesis of Sjögren’s syndrome and psoriasis, leading to glandular damage and psoriatic skin, respectively (20, 84–87).

**Figure 2:**
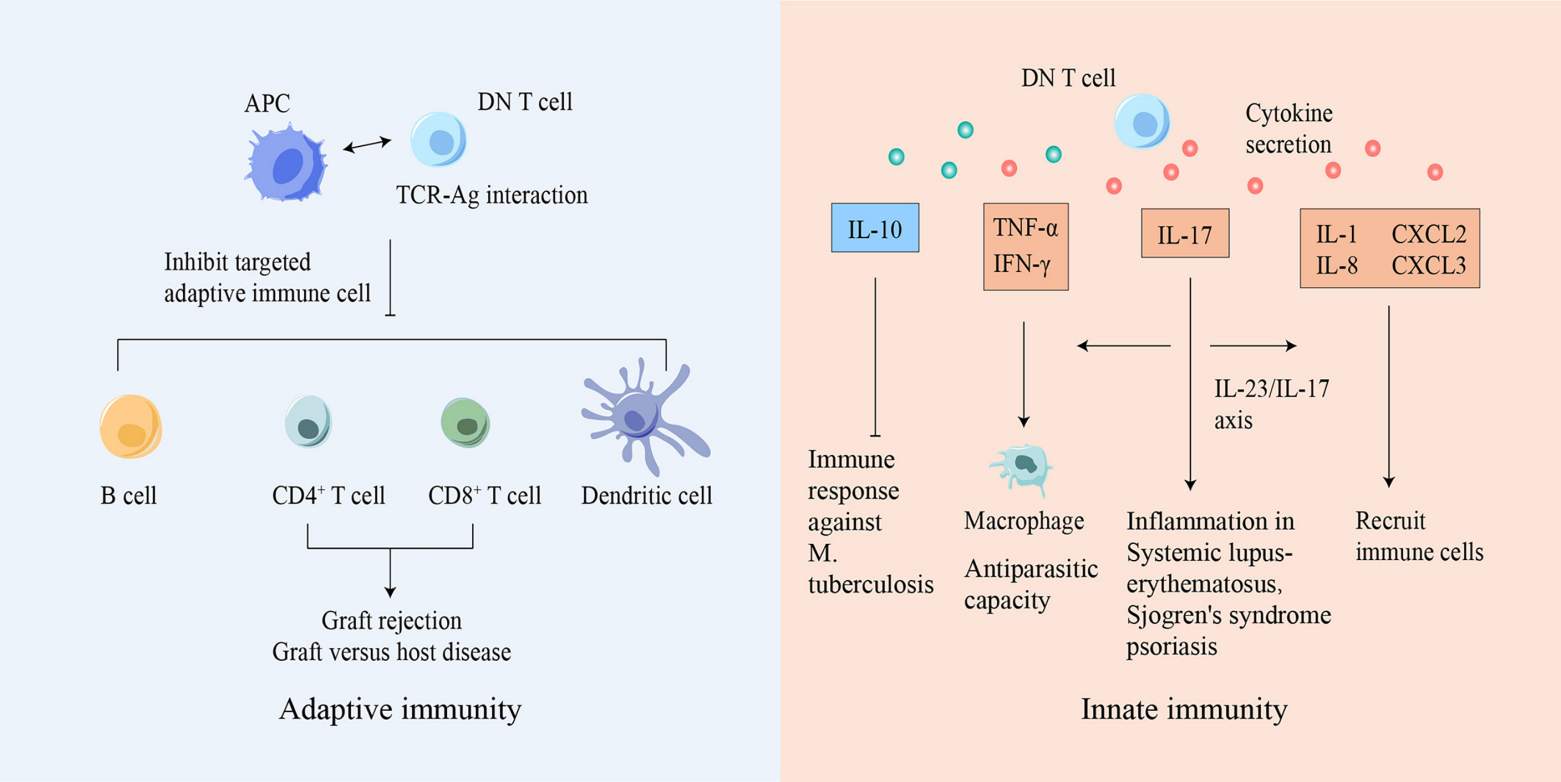
DN T cells participate in both innate and adaptive immunity. Based on Ag-TCR-specific recognition, DN T cells can inhibit or eliminate adaptive immune cells, suppressing diseases caused by immune responses. DN T cells can also secrete various cytokines to mediate innate immune responses. Ag, antigen; APC, antigen presenting cell; CXCL, chemokine ligand; DN, double-negative; IFN-γ, interferon-γ; IL, interleukin; M. tuberculosis, Mycobacterium tuberculosis; TCR, T cell receptor; TNF, tumor necrosis factor.

Based on these studies, we conclude that DN T cells participate in immune surveillance, defense, and homeostasis (**Table 2** and **Figure 2**). More studies in disease models are urgently needed to understand the critical roles of DN T cells and the underlying mechanisms in specific settings, which may facilitate diagnosis and therapy.

**Table 2:** T helper-like DN T cell in response to inflammation.

Mouse model or human	DN T cell phenotype	Pro/anti-inflammatory profile and possible mechanism	Promote/inhibit disease progress	Reference
Mice infected with F. tularensis	TCRαβ^+^	IL-17A in early response, IL-17A synergizes with IFN-γ to inhibit LVS growth in alveolar epithelial cells and macrophages	Inhibit	(72)
Non-severe M. tuberculosis-infected individuals	TCRαβ^+^/γδ^+^CD69^high^ HLA-DR^high^	IFN-γ, TNF-α, critical for host control of M. tuberculosis	Inhibit	(77)
Severe M. tuberculosis-infected individuals	TCRγδ^+^CD69^high^ HLA-DR^high^	IL-10, inhibit immune response against M. tuberculosis	-	(77)
Healed Leishmania-infected C57BL/6 (Thy1.2) mice	TCRαβ^+^CD62L^high^ CD44^high^	IFN-γ, TNF, secondary immune response	Inhibit	(73)
Leishmania-infected individuals	TCRαβ^+^CD25^high^ CD28^high^CD56^high^ CD69^high^ CD45RO^high^ HLA-DR^high^	IFN-γ, TNF-α	Inhibit	(76)
natural non-human hosts of SIV	TCRαβ^+^CD3^+^CD28^+^	IFN-γ, TNF-α, IL-4, IL-17, compensate for low CD4^+^ T cells	Inhibit	(8)
ALPS patients	TCRαβ^+^B220^+^CD27^+^CD28^+^	Defects in Fas-mediated apoptosis, accumulation of DN T cells	Promote	(35)
SLE patients	TCRαβ^+^	IL-23 receptor, ROR-γt, IL-17, similar to IL-23/Th17 axis	Promote	(9)
Sjögren’s syndrome patients	TCRαβ^+^	IL-17	Promote	(85)
Psoriatic skin inflammation in K14.Stat3C transgenic mice	TCRαβ^+^CCR6^+^	IL-17, ROR-γt, similar to IL-23/Th17 axis	Promote	(87)
Psoriasis patients	TCRαβ^+^CD45RA^+^ CCR7^-^	IFN-γ, Th-1 cytokine, induction of IL-17 expressing T cells and T cell recruitment to inflamed tissues	Promote	(86)
Healthy human	-	CXCL3, CXCL2, IL-1, IL-8, IL-10	-	(9)

αβ, alpha and beta; ALPS, autoimmune lymphoproliferative syndrome; CXCL, chemokine ligand; DN, double-negative; F. tularensis, Francisella tularensis; γδ, gamma and delta; IFN-γ, interferon-γ; IL, interleukin; LVS, live vaccine strain; M. tuberculosis, Mycobacterium tuberculosis; ROR-γt, retinoic acid-related orphan receptor γt; SIV, simian immunodeficiency virus; SLE, systemic lupus erythematosus; TCR, T cell receptor; Th-1, T-helper type 1; TNF, tumor necrosis factor.

## The Warrior and Culprit in Cancer Development

It is well known that the immune system plays a vital role in the development and progression of cancer. DN T cells exist as an essential T cell subset that plays an indispensable role in immune system function. Many studies have explored the role of DN T cells in various types of tumors and found that DN T cells possess protumor or antitumor effects depending on the tumor type. In addition, aberrant DN T cells have also been detected within lymphoma.

### The Role of DN T Cells During Cancer Development

**Figure 3:**
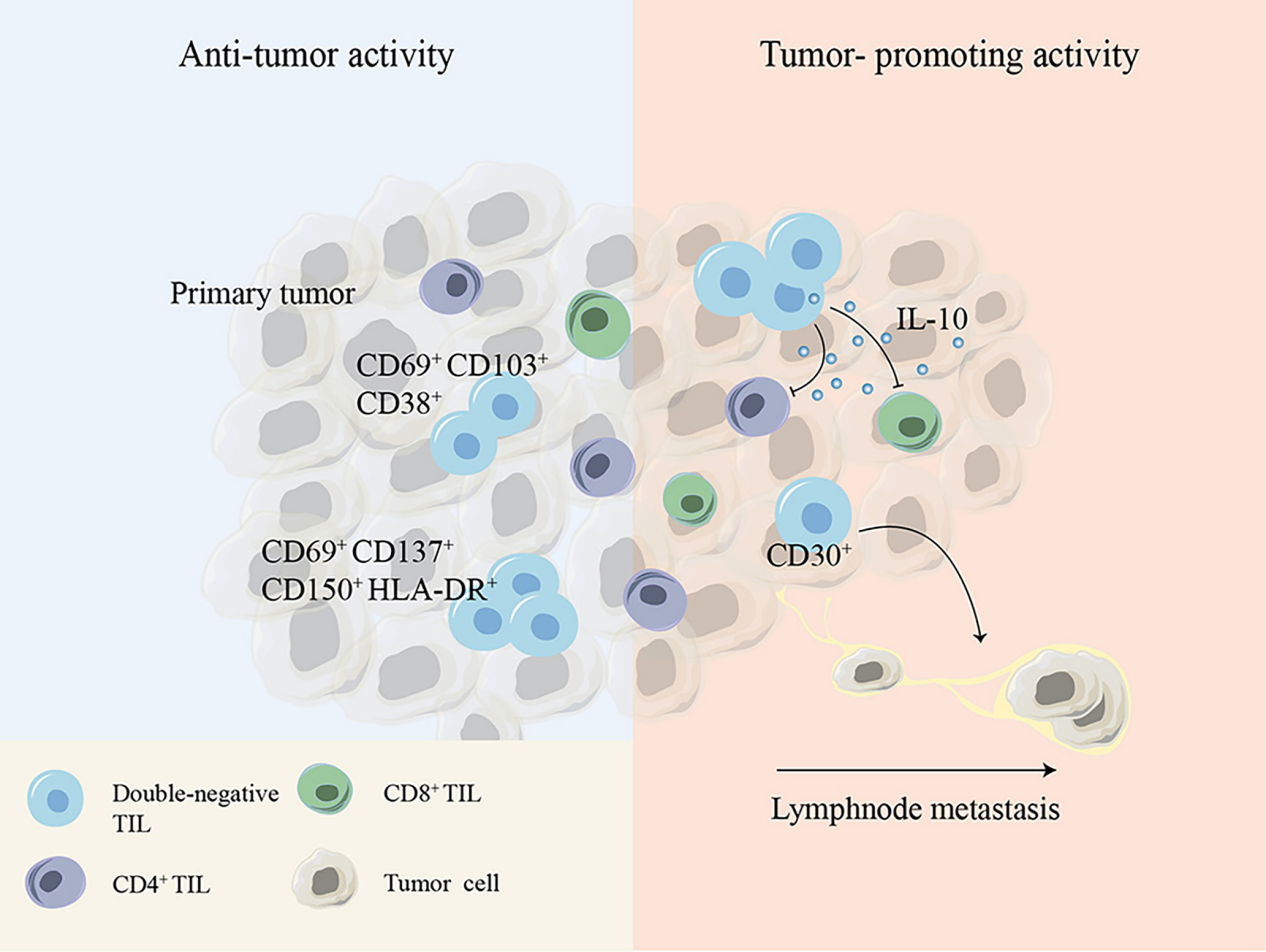
Cell surface molecules or cytokines expressed by DN TILs show debatable antitumor or tumor-promoting activity during tumor development. DN, double-negative; TIL, tumor-infiltrating lymphocyte.

DN T cells have been shown to not only exist in the peripheral blood but also infiltrate solid tumors such as non-small-cell lung cancer (NSCLC), liver cancer, glioma and pancreatic tumors (10, 88–90). It has been reported that TCRαβ^+^ DN T cells account for 1.4% of CD3^+^ T cells within the tumor tissues of NSCLC patients (91). Immune profiling of hepatocellular carcinoma (HCC)-infiltrating cells showed two unique populations of DN T cells: one cluster of DN T cells is TCRγδ^+^, coexpressing the tissue-resident markers CD69 and CD103 and the activation marker CD38, and the other cluster is TCRαβ^+^, with expression of the activation markers CD150, CD69, CD137 and HLA-DR and the inhibitory TIGIT molecule. Although the details of the Ag-presenting process remain unknown, the expression of activation markers suggests that DN tumor-infiltrating lymphocytes (TILs) might be activated within HCC (88). Another study found a group of major defective TCRαβ^+^ DN T cells that secrete IL-10 with suppressive potential within murine glioma and melanoma. Decreasing the number of DN TILs by disruption of the *fyn* gene markedly improved host survival without affecting other TILs, demonstrating the immunosuppressive functions of TCRαβ^+^ DN TILs in murine glioma and melanoma models (92). The presence of DN TILs has also been demonstrated within lymph node metastases of melanoma patients with tolerogenic behavior biomarkers such as CD30, and a significant increase in DN TILs was found in lymph nodes of patients with melanoma who had experienced disease progression compared to that in patients without disease progression, leading to the hypothesis that DN TILs contribute to tumor metastatic progression (93, 94) (**Figure 3**).

These controversial conclusions indicate that DN T cells play either tumor-promoting or tumor-inhibiting effects depending on the distinct tumor microenvironment and tumor type (**Figure 3**).

### DN T Cell Associated-Cancerous T Cell Lymphoma

With accumulation of genetic and epigenetic alterations, every single cell possesses the possibility to become cancerous, and DN T cells seem to be no exception. To date, a series of studies have shown that this rare T cell subset can cause lymphoma.

As a collection of non-Hodgkin lymphoma subtypes, cutaneous T cell lymphoma (CTCL) remains a rare malignancy, and the exact pathogenesis is still unknown. Unlike most non-Hodgkin lymphomas, which are generally B cell-related, CTCL is caused by mutations in T cells, and more than half of the cases include mycosis fungoides (MF), which is characterized by widely distributed ulceration, superficial crusts, necrosis or hemorrhage. Clinically, DN cell lymphoma patients have otherwise classic features of MF, including hypopigmented patches and plaques. Eighteen (12%) MF patients had CD3^+^CD4^-^CD8^-^ MF cells in 140 patients with early-stage MF, indicating that this cell subtype is not rare in early MF. Similar to CD4^+^ MF cells, most DN cells express TCRαβ and the memory marker CD45RO; however, unlike CD4^+^ MF cells, most DN cells express the cytotoxic molecule TIA-1, and this aberrant phenotype of DN cells does not seem to be associated with prognostic significance (95). A case report by Kempf et al. showed that intraepidermal DN lymphocytes are negative for TIA-1, granzyme B and perforin but positive for programmed cell death protein 1 (PD-1) (96). More recently, Miyauchi et al. reported a case of DN CTCL with clinical and pathological characteristics similar to those of primary cutaneous CD8^+^ CTCL, characterized by TCRβ positivity and expression of granzyme B, perforin and cytotoxic marker TIA-1 (97). Additionally, Beltran et al. reported a case of peripheral T cell lymphoma characterized by neoplastic DN Treg cells that were positive for CD3, CD25 and Foxp3 but negative for CD4 (98).

Although some conclusions came from case reports, these studies provide insight into the specific heterogeneous phenotypes of DN non-Hodgkin lymphoma. Further exploration is needed to determine whether different phenotypes are associated with disease progression or prognosis.

## DN T Cells in Tumor Immunotherapy

Recently, tumor immunotherapies have achieved remarkable successes in clinical use. Current tumor immunotherapy strategies can be briefly divided into 5 main groups: immune checkpoint inhibitors, adoptive cell therapy (ACT), monoclonal antibodies, vaccines and immune system modulators. ACT mainly includes tumor-infiltrating lymphocyte therapy, engineered TCR therapy, chimeric Ag receptor T cell therapy and NK cell therapy.

### DN T Cells as Independent Antitumor Agents

#### Role of DN T Cells in Treating Hematological Malignancies

Immunotherapy has been an incredibly promising treatment for hematologic malignancies in recent years, and DN T cells were first developed as a viable antitumor therapy in acute myeloid leukemia (AML). AML patient-derived DN T cells could effectively target both allogeneic and autologous AML cells. Compared to *ex vivo*-expanded CD8^+^ T cells and NK cells, DN T cells possess higher cytotoxicity toward allogeneic leukemic cells, indicating that they could be a therapy for AML (11). More advanced, healthy donor-derived DN T cells also exert cytotoxicity against allogeneic AML cells, including chemotherapy-resistant leukemic cells, in a DN T cell donor-unrestricted manner (99).

**Figure 4:**
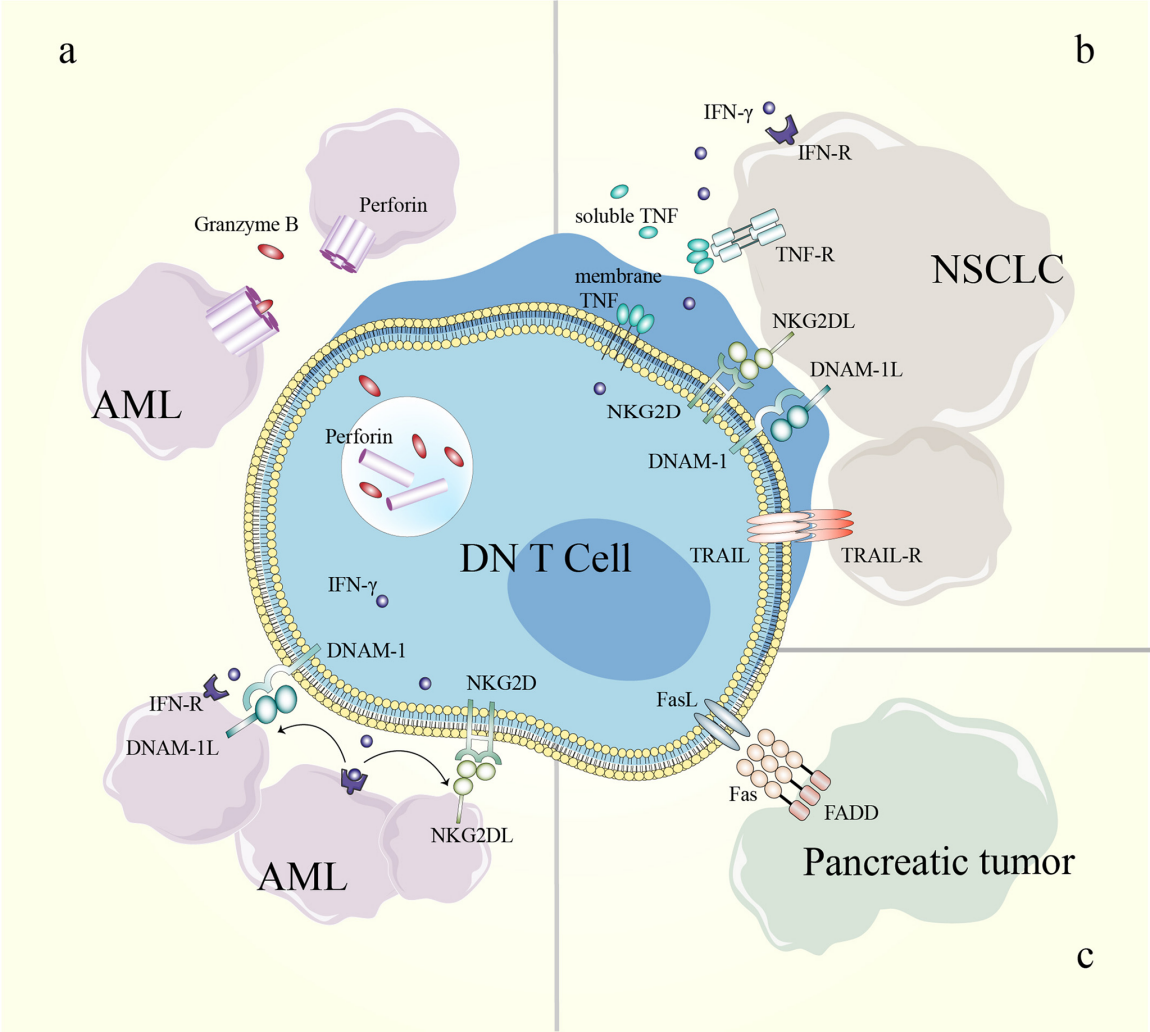
Mechanisms of DN T cells in cancer treatment. **(A)** The killing effect of DN T cells toward AML cells can be perforin/granzyme B-dependent; IFN-γ enhances the effect of NKG2D and DNAM-1 in the DN T cell-mediated inhibition of AML cells. **(B)** NKG2D, DNAM-1 and soluble TRAIL participate in DN T cell-mediated lysis of NSCLC cells. **(C)** DN T cells eliminate pancreatic tumor cells through the Fas/FasL pathway. AML, acute myeloid leukemia; DN, double-negative; DNAM-1, DNAX accessory molecule-1; DNAM-1L, DNAX accessory molecule-1 ligand; FADD, Fas-associating protein with a novel death domain; FasL, Fas ligand; IFN-γ, interferon-γ; IFN-R, interferon receptor; NKG2D, natural-killer group 2, member D; NKG2DL, natural-killer group 2, member D ligand; NSCLC, non-small-cell lung cancer; TNF, tumor necrosis factor; TNF-R, tumor necrosis factor receptor; TRAIL, TNF-related apoptosis-inducing ligand; TRAIL-R, TNF-related apoptosis-inducing ligand receptor.

Mechanistically, DN T cells preferentially recognize and target AML cells in a human leukocyte Ag-independent and TCR-unrestricted manner. *Ex vivo*-expanded DN T cells express high levels of IFN-γ, TNF-α, perforin, granzyme B and natural cytotoxic receptors. AML patient-derived DN T cells target leukemia cells through a perforin-dependent pathway, while healthy donor-derived DN T cells kill leukemia cells partly through IFN-γ, NKG2D and DNAX accessory molecule-1 (DNAM-1). Furthermore, IFN-γ exerts positive feedback by increasing the expression of ligands for NKG2D and DNAM-1 on AML cells. The difference is possibly caused by the heterogeneity of DN T cells among different donors (11, 100) (**Table 3** and **Figure 4**). In a recent study, genes that determine the susceptibility of AML cells to DN T cell therapy were identified using a CRISPR/Cas9 screen. The results showed that components of the Spt-Ada-Gcn5-acetyltransferase complex and the molecule CD64 are potential biomarkers for DN T cell therapy, and activation of CD64 might help improve the efficacy of DN T cell therapy in AML (103).

**Table 3:** Anti-tumor DN T cell in cancer treatment.

DN T cell source	TCR Type	Mechanism of Cytotoxicity	Target Cell	Environment	Reference
AML patients in CR	αβ^+^TCR and γδ^+^TCR	Highly perforin dependent pathway, Ag-nonspecific	AML cell	*Ex vivo*	(11)
*Ex vivo* expanded from healthy human peripheral blood	αβ^+^TCR and γδ^+^TCR	NKG2D/NKG2DL, DNAM-1/DNAM-1L, positive loop interaction between IFN-γ and NKG2D or DNAM-1	AML cell	Patient-derived xenograft model	(100)
	-	Fas/FasL pathway	Pancreatic tumor cell	Cell line-derived xenograft model	(101)
	-	IFN-γ, TNF-α	NSCLC cell	Cell line-derived xenograft model	(10)
	γδ^+^TCR	NKG2D, DNAM-1, NKp30 and TRAIL	NSCLC cell	Cell line-derived xenograft model	(102)

αβ, alpha and beta; AML, acute myeloid leukemia; CR, complete response; DN, double-negative; DNAM, DNAX accessory molecule-1; DNAM-1L, DNAX accessory molecule-1 ligand; FasL, Fas ligand; γδ, gamma and delta; IFN-γ, interferon-γ; NSCLC, non-small-cell lung cancer; NKG2D, natural-killer group 2, member D; NKG2DL, natural-killer group 2 ligand; TNF-α, tumor necrosis factor; TRAIL, TNF-related apoptosis-inducing ligand.

In summary, both patient- and healthy donor-derived allogeneic DN T cells can induce cytotoxicity against AML cells through contact and noncontact pathways. Analyzing the gene profile of AML might help distinguish the susceptibility of AML to DN T cell therapy.

#### Role of DN T Cells in Solid Tumors

Due to the complex microenvironment and difficulties of delivery, the efficiency of immune cell therapy is limited in solid tumors. However, an increased understanding of the antitumor effects of DN T cells in pancreatic cancer and NSCLC has been gained in recent years (10, 101, 102, 104). DN T cells effectively inhibit pancreatic tumor growth through the Fas/FasL pathway in patient-derived xenograft models. In detail, the binding of FasL induces receptor oligomerization, which leads to the recruitment and activation of caspase-8, which further induces apoptosis *via* caspase-3-induced Bid cleavage (105–107). Adoptive transfer of healthy donor-derived DN T cells also inhibits the growth of NSCLC xenografts and prolongs xenograft survival in recipient mice (10, 102). DN T cell-mediated lysis of NSCLC cells relies on the expression of innate receptors (NKG2D, DNAM-1 and NKp30) and soluble TNF-related apoptosis-inducing ligand (TRAIL) in DN T cells. In addition, the heterogeneity in the susceptibility of NSCLC cells to DN T cell lysis depends in part on the different expression levels of cognate ligands in NSCLC cell lines (102) (**Table 3**) (**Figure 4**). Thus, a better understanding of the expression patterns might help guide the selection of patients who will respond to DN T cell therapy.

### Combined Therapy With DN T Cells

Combination therapy is a vital treatment strategy for malignant tumors, with potential synergistic antitumor effects and decreased side effects. Li Zhang’s group found that DN T cells combined with chemotherapy, immune checkpoint blockade or immunomodulatory cytokines exhibit synergistic antitumor effects against hematological malignancies and solid tumors (10, 99, 102, 108).

Chemotherapy is the main treatment strategy for tumors, but intrinsic and adaptive resistance to chemotherapy limit the outcome of tumor therapy. As chemotherapeutic agents can regulate the tumor microenvironment, chemotherapy plus DN T cells have been proven to be effective for AML. Chen et al. reported that DN T cells combined with the chemotherapeutic drug Daunorubicin efficiently kill chemotherapy-resistant primary AML cells, producing synergistic effects, and these effects are induced by increased expression of NKG2D and DNAM-1 ligands on leukemia cells by Daunorubicin (99). Furthermore, NKG2D ligands can be upregulated and subsequently induce the activation of the downstream transducer kinase ATM in the DNA damage response (109). Whether Daunorubicin-induced NKG2D ligand expression shares the same signaling pathway needs to be determined in further explorations. In newly diagnosed AML patients who are unfit for conventional intensive chemotherapy, Venetoclax combined with Azacytidine has achieved remarkable clinical outcomes (110, 111), and recently, Lee et al. discovered that Venetoclax can enhance DN T cell-mediated cytotoxicity against AML cells by increasing reactive oxygen species generation and subsequently increasing NKG2D and DNAM-1 expression in DN T cells. In addition, Azacytidine increased the susceptibility of AML cells to DN T cells by activating the STING/cGAS pathway and inducing a viral mimicry response. In conclusion, combination therapy with DN T cells and traditional drugs can achieve synergistic effects (108).

DN T cells combined with immune checkpoint blockade also show enhanced antitumor effects compared to single therapy. Fang et al. showed that over 50% of DN TILs from NSCLC patients express PD-1. Blocking PD-1 induce resistance of tumor-mediated inhibition of DN T cell infiltration, augment NKG2D^+^ and DNAM-1^+^ DN T cells and induce cytolytic granule secretion in NSCLC xenografts (10). Thus, DN T cells combined with immune checkpoint blockade therapy may be ideal for patients with “immune desert” tumors who are resistant to anti-PD-1 therapy.

DN T cells combined with immunomodulators provide promising alternatives for NSCLC treatment. Yao et al. reported that IL-15 augments the killing ability of DN T cells (primarily TCRγδ^+^ DN T cells) in primary and established NSCLC cell lines. The expression of NKG2D and NKp30 and secretion of soluble TRAIL by DN T cells increase significantly with additional IL-15 stimulation, which is responsible for DN T cell-mediated NSCLC cytolysis (102). Therefore, IL-15 enhances the DN T cell-killing effect in a wide spectrum of NSCLC cell lines.

In conclusion, the combination of DN T cells and conventional therapies has exhibited enhanced cytolysis in preclinical studies, and these combined strategies are expected to be translated into the clinic in the future.

### Advantages of DN T Cell Therapy in Preclinical and Clinical Applications

ACT provides the promise of a powerful option for tumor treatment, especially for hematological malignancies. Although chimeric Ag receptor-modified T cell therapy is one of the most promising ACTs applied clinically, it still has challenges, including complications and technical and economic issues. Here, we summarize the reasons why DN T cell therapy is likely to earn a place in ACT.

#### High Technical Feasibility and Convenience

Technical feasibility dictates the ease of translation from theory to application, and DN T cells for therapy has been proven to be easily acquired, expanded and preserved, therefore serving as an off-the-shelf form of ACT. Li Zhang’s team has successfully expanded large-scale AML patient-derived DN T cells and healthy donor-derived DN T cells to a therapeutic number under good manufacturing practice conditions. In detail, from 1 ml of peripheral blood, 1.41 ± 0.51 × 10^8^ DN T cells were obtained only 17-20 days after *ex vivo* expansion with over 90% purity (11, 100). Moreover, human DN T cells maintain comparable viability and antileukemia ability even after cryopreservation (112). Collectively, these data support the use of DN T cells as an off-the-shelf ACT for tumor therapy.

#### Potent Anticancer Effectiveness and Safety

Preclinical data have shown that allogeneic healthy human-derived DN T cells exert diverse antitumor activities toward T cell leukemia, B cell lymphoma, AML, lung cancer and pancreatic cancer in TCR-independent, human leukocyte Ag-unrestricted and donor-unrestricted manners (100–102, 104, 112). Notably, DN T cells have more cytotoxic activity toward leukemic cells than do primary CD4^+^ T cells, CD8^+^ T cells and NK cells (11, 100). In addition, researchers also conducted safety assessments of DN T cell therapy. For allogeneic cell therapy, GVHD is the most serious and inevitable complication. Aside from the inhibitory effect of DN Treg cells mentioned above, allogeneic DN T cells do not cause GVHD and have no cytotoxicity toward normal peripheral blood mononuclear cells, hematopoietic stem cells, progenitor cells, or peripheral blood myeloid cells in xenograft models. Despite infiltrating the intestines, liver and lungs, DN T cells do not cause tissue damage in xenograft models. In addition, human DN T cells were found to persist *in vivo* for at least four weeks in the presence of allogeneic conventional T cells, confirming that allogeneic DN T cells are resistant to host-versus-graft reactions (112). Taken together, these results demonstrate that anticancer allo-DN T cell therapy is relatively safe and tolerable.

Recently, a first-in-human phase I/IIa clinical trial using allogeneic DN T cells to treat patients with relapsed AML after allogeneic hematopoietic stem cell transplantation was successfully conducted. An overall initial response rate of 63.6% (7/11) was achieved within 11 evaluable patients (6 achieved complete remission, and 1 achieved partial remission). The 1-year overall survival after DN T cell treatment was 45.5%. Notably, none of the patients has developed GVHD or experienced adverse events higher than grade 2 thus far, while all patients experienced grade 1 or 2 cytokine release syndrome and then recovered quickly. Thus, this clinical study showed that allo-DN T cell therapy is safe and tolerable for AML patients, and an expanded patient cohort is urgently required for the next phase of the clinical trial (113, 114).

#### Potential Limitations

As a novel ACT, DN T cells also face potential challenges. DN T cells exhibit antitumor properties, but there is no evidence that they could completely eradicate tumors; thus, DN T cell therapy might induce partial remission rather than complete remission. Although human DN T cells can persist *in vivo* for several weeks (111), once DN T cells cannot be detected *in vivo*, reinfusion of cryopreserved DN T cells might be needed, which means that the interval and number of cycles for DN T cell reinfusion need to be investigated. In addition, many findings of human DN T cells are from studies in immunodeficient mice, but the tumor microenvironment inside patients may differ greatly. To date, only one clinical trial has shown that allo-DN T cell therapy is safe and tolerable for AML patients, and further clinical trials with a larger study population and a broad range of tumor types, including solid tumors, still need to be performed (114).

## Conclusion

**Figure 5:**
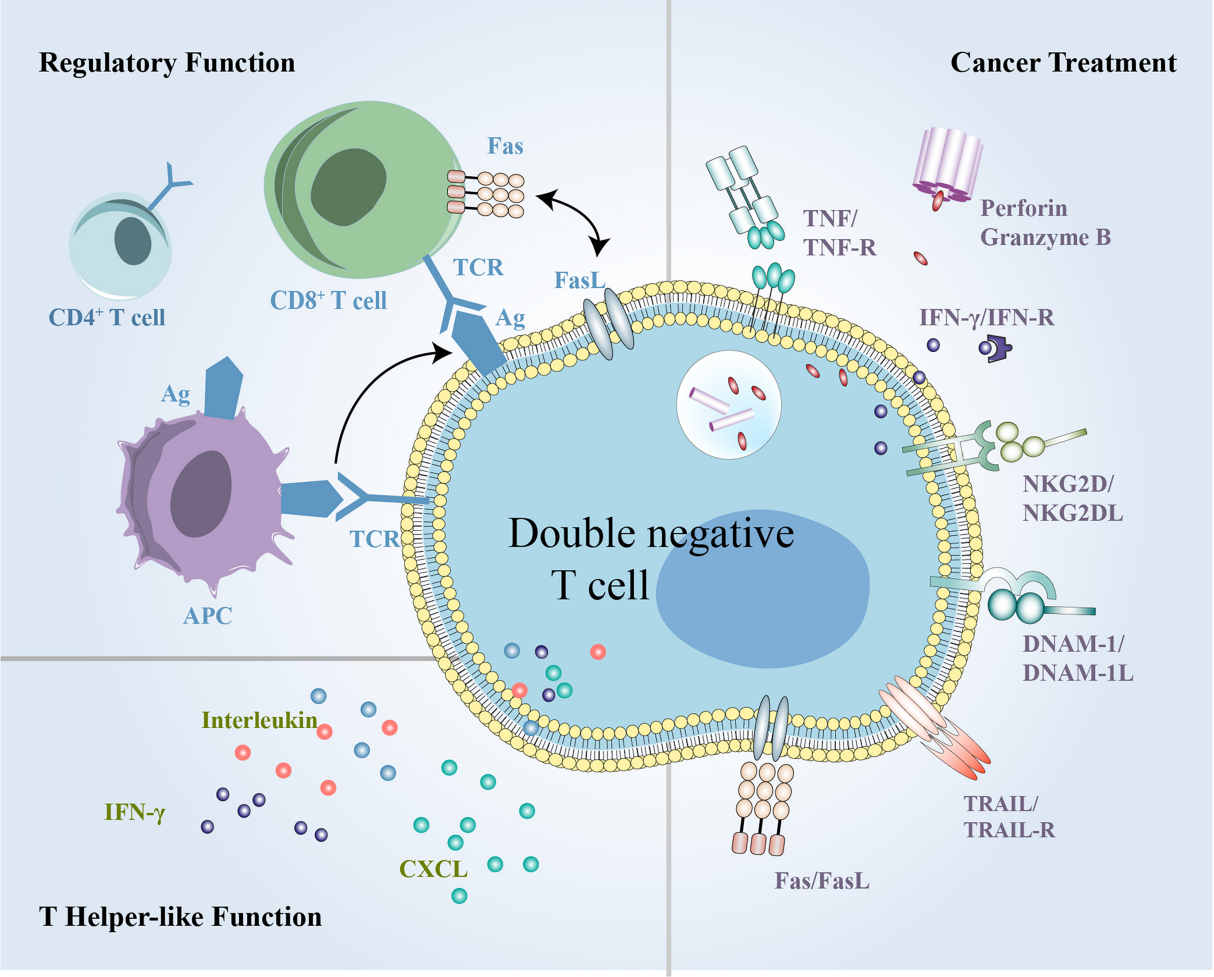
The summary of multifunctional DN T cells in inflammation, immune disorders and cancer. DN T cells exert both regulatory and helper-like functions under different conditions and can exert antitumor activity in cancer treatment. DN, double-negative.

DN T cells, an important subset of mature T lymphocytes, exist as a rare population in peripheral blood and tissues and play a vital role in immune homeostasis under healthy conditions. In addition, they act as Treg cells, cytotoxic T cells or Th cells and influence innate and adaptive immune systems in distinct pathological conditions, which are closely linked with inflammatory disease, autoimmune disease, tumorigenesis and tumor development. In addition, as an off-the-shelf ACT, adoptive DN T cell therapy has emerged as an ideal therapeutic option for tumors, and DN T cells exhibit cytotoxicity in various hematological malignancies and solid tumors in an Ag-independent manner (**Figure 5**). In conclusion, by summarizing research findings, we have realized that the heterogeneity and various functions of DN T cells and the use of DN T cells as a potential therapeutic tool need to be further explored in the era of precision and personalized medicine.

## Author Contributions

ZW, HP, and JY conceived of the manuscript. ZW wrote the manuscript and created the figures. JS, YZ, YH, YY, and JY edited the manuscript and figures. All authors contributed to the article and approved the submitted version.

## Funding

JY is supported by grants from the National Natural Science Foundation of China (81803070). JS is supported by grants from the National Natural Science Foundation of China (81702809). YZ is supported by grants from Zhejiang Provincial Natural Science Foundation of China (LSY19H160006).

## Conflict of Interest

The authors declare that the research was conducted in the absence of any commercial or financial relationships that could be construed as a potential conflict of interest.

## Publisher’s Note

All claims expressed in this article are solely those of the authors and do not necessarily represent those of their affiliated organizations, or those of the publisher, the editors and the reviewers. Any product that may be evaluated in this article, or claim that may be made by its manufacturer, is not guaranteed or endorsed by the publisher.

## Abbreviations

αβ, alpha and beta; ACT, adoptive cell therapy; Ag, antigen; ALPS, autoimmune lymphoproliferative syndrome; AML, acute myeloid leukemia; CTCL, cutaneous T cell lymphoma; DN, double-negative; DNAM-1, DNAX accessory molecule-1; DP, double-positive; GVHD, graft-versus-host disease; γδ, gamma and delta; HCC, hepatocellular carcinoma; HIV, human immunodeficiency virus; IFN-γ, interferon-γ; IL, interleukin; MF, mycosis fungoides; MHC, major histocompatibility complex; NK, natural killer; NKG2D, natural-killer group 2, member D; NSCLC, non-small-cell lung cancer; Ox40, tumor necrosis factor receptor superfamily, member 4; PD-1, programmed cell death protein 1; SLE, systemic lupus erythematosus; SP, single-positive; Th, T-helper; TILs, tumor-infiltrating lymphocytes; TNF-α, tumor necrosis factor-α; TRAIL, TNF-related apoptosis-inducing ligand; Treg, regulatory T.
